# Evaluation of Selective Efficacy of Indocyanine Green-Mediated Photodynamic Therapy ICG-PDT in MCF-7 Breast Cancer Cells Compared to Healthy Cells in a 3D Hollow Fiber Bioreactor Model

**DOI:** 10.3390/ph18121832

**Published:** 2025-12-01

**Authors:** Wiktoria Mytych, Magdalena Czarnecka-Czapczyńska, Dorota Bartusik-Aebisher, David Aebisher, Gabriela Henrykowska, Aleksandra Kawczyk-Krupka

**Affiliations:** 1 Department of Photomedicine and Physical Chemistry, Medical College, The Rzeszów University, 35-310 Rzeszów, Poland; wiktoriamytych@gmail.com; 2 Department of Internal Diseases, Angiology and Physical Medicine, Center for Laser Diagnostics and Therapy, Medical University of Silesia, 41-902 Bytom, Poland; magdalena.czarnecka921114@gmail.com (M.C.-C.); akawczyk@gmail.com (A.K.-K.); 3 Department of Biochemistry and General Chemistry, Medical College, The Rzeszów University, 35-310 Rzeszów, Poland; dbartusikaebisher@ur.edu.pl; 4 Department of Epidemiology and Health Public, Faculty of Medicine, Medical University in Łodz, 90-419 Lodz, Poland

**Keywords:** photodynamic therapy, indocyanine green, breast cancer, MCF-7

## Abstract

**Objective:** This study investigates the efficacy of indocyanine green-mediated photodynamic therapy (PDT) in targeting MCF-7 breast cancer cells, a representative model of luminal A subtype, compared to healthy breast epithelial cells. **Methods:** MCF-7 cells and healthy breast cells were cultured in a three-dimensional (3D) hollow fiber bioreactor to mimic the tumor microenvironment in vivo. Cells were treated with ICG at concentrations ranging from 1 to 1000 μM and then photoactivated using a diode laser. Cell viability was assessed by trypan blue staining, and the production of reactive oxygen species (ROS), including singlet oxygen (^1^O_2_) was measured. **Results:** Cell viability, assessed via trypan blue exclusion, decreased dose-dependently with ICG concentrations (1–1000 μM), with MCF-7 viability dropping from 94.5% ± 0.8% at 0.1 μM to 15.83% ± 0.66% at 1000 μM, compared to healthy cells retaining >50% viability up to 500 μM (55.2% ± 2.0% at 1000 μM). Student’s t-tests confirmed significant differences (*p* < 0.05) between MCF-7 and control (0 μM) at all concentrations, and between MCF-7 and healthy cells, indicating selective cytotoxicity (IC_50_: ~75 μM for MCF-7). Flow cytometry revealed MCF-7 cell concentrations were significantly lower than healthy cells’ across all ICG doses and seeding densities (*p* < 0.05). Spectroscopic analyses showed ICG absorption peaks at 800–900 nm, fluorescence at 800–820 nm, and singlet oxygen phosphorescence at 1270 nm, confirming effective ROS generation. **Conclusions:** Cell concentrations confirmed selective MCF-7 cytotoxicity (*p* < 0.05). Spectroscopic data validated ROS generation, supporting ICG-PDT’s potential as a selective therapy for early-stage breast cancer within a 50–500 μM therapeutic window.

## 1. Introduction

**Figure 1:**
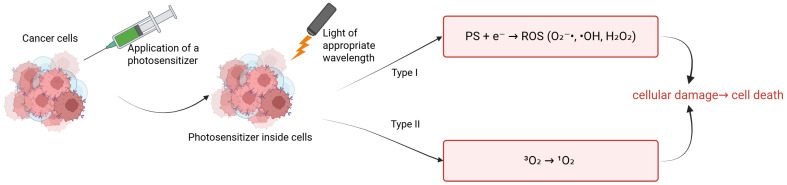
Types of reactions in PDT.

Breast cancer remains one of the most diagnosed malignant cancers in the world, posing a serious challenge to modern oncology [1]. According to the World Health Organization (WHO), more than 2.3 million new cases of breast cancer were reported in 2022, highlighting the need for new, effective therapeutic strategies [2]. The MCF-7 cell line, isolated in 1970 from the pleural effusion of a 69-year-old patient with metastatic breast cancer, is one of the most important in vitro models used in breast cancer research [3]. It expresses estrogen receptors (ER), progesterone receptors (PRs), and moderate levels of HER2, making it representative of luminal subtype A of breast cancer [4]. The MCF-7 cell line is widely used to study the mechanisms of proliferation, apoptosis, and response to various forms of therapy, including chemotherapy, radiotherapy, and novel approaches such as photodynamic therapy (PDT) [5,6]. Indocyanine green (ICG) is an FDA-approved fluorescent dye that is currently used in diagnostic imaging and PDT [1]. PDT consists of three basic components: a photosensitizer (PS), singlet oxygen, and light. The mechanism of PDT is based on administering a photosensitizer that accumulates in the desired cells and then influencing these cells with light of the appropriate PS wavelength under aerobic conditions [2]. These mechanisms lead to the formation of reactive oxygen spices (ROS) that induce target cell death. The PS can be administered locally or systemically [3]. There is an accumulation of PS in the desired tissue and then absorption of the light energy of the applied light. The PS transitions from the ground state (S_0_) to the excited singlet state (S_1_), and then through the intersystem transition to the long-lived triplet state (T_1_). In the T_1_ state, the PS can donate an electron or hydrogen atom, leading to the formation of free radicals (type I) such as superoxide anion radicals (O_2_^−^), hydroxyl radicals (-OH), or hydrogen peroxide (H_2_O_2_), or transfer energy directly to the oxygen molecule, thereby producing singlet oxygen (^1^O_2_) (type II) [4]. Singlet oxygen has a high reactivity that leads to damage to lipids, nucleic acids, or proteins at the cellular level. This leads to the activation of apoptosis, necrosis, or autophagy [5]. Both types I and II (Figure 1) can occur simultaneously. However, the dominance of one type is dependent on the main components of PDT [6]. In type I, the mechanism of the PS in the triplet state involves transferring an electron or hydrogen atom to the substrates and thus free radicals are formed. In type II, singlet oxygen is produced, the energy of which is transferred directly to molecular oxygen, thus creating singlet oxygen [7]. In MCF-7, PDT leads to dominant apoptosis via ROS, activating caspases and damaging mitochondria.

**Figure 2:**
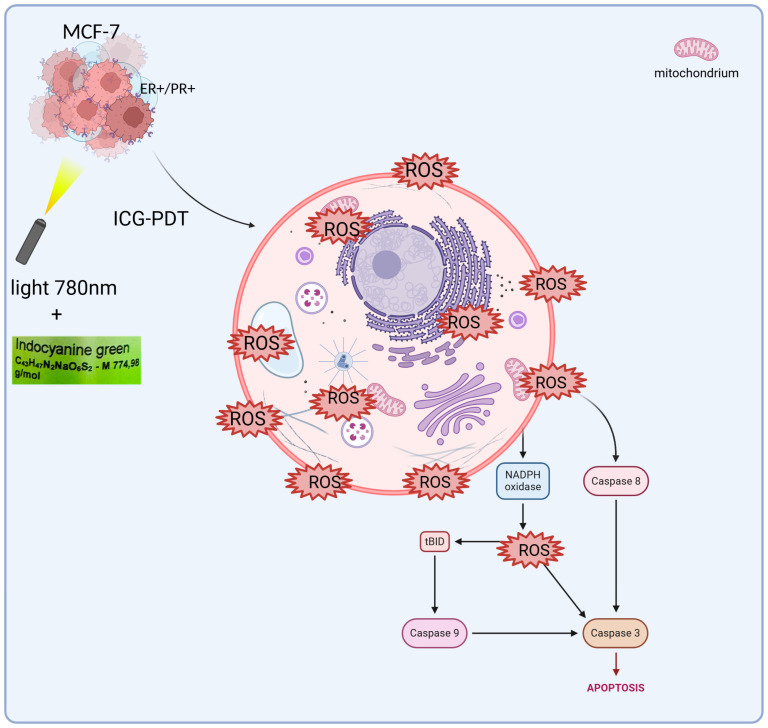
ICG-PDT in MCF-7 cells. This process begins with the cellular uptake of ICG. Then, when exposed to light (hv), reactive oxygen species (ROS) are generated by NADPH oxidase. ROS activate caspase 8, which in turn activates tBID, leading to the release of further signals in the mitochondria. This triggers caspase 9 and then caspase 3, leading to apoptosis.

ICG cell damage is exacerbated when activated by near-infrared light (NIR). Energy transfer to oxygen molecules and activation of ROS. ICG absorbs light at ~780 nm to enter an excited state in a 3D breast cancer cell culture medium, enabling fluorescence emission and energy transfer for PDT [8]. A conversion to an excited triplet state takes place. Intravenous ICG is bound to plasma proteins, mainly albumin. It is taken up by hepatocytes and removed only by the liver along with bile [9]. ICG does not change its form and does not undergo metabolism or hepatointestinal circulation. This therefore means that the functionality of hepatocytes is crucial for the half-life and elimination of ICGs from the body [10]. ICG is usually well tolerated by patients; in some cases it can cause anaphylactic reactions. It is generally considered safe to use PSs [11]. In breast cancer studies, particularly using the MCF-7 lineage (Figure 2), ICG has been shown to cause molecular changes.

The Bcl-2 protein, which protects cells from apoptosis after PDT treatment, decreases, causing cancer cells to die. On the other hand, PDT increases the level of Bax protein, which promotes apoptosis [9,12,13,14,15,16,17,18]. Studies on MCF-7 confirm that PDT induces apoptosis through the activation of caspase-3 in the late stages, with a simultaneous decrease in Bcl-2 and an increase in Bax. In MCF-7 PDT with hypericin conjugated to AuNPs induces apoptosis through an increase in Bax and a decrease in Bcl-2, synergizing with ROS as in ICG-PDT. It also could act in the G_1_ phase of the cell cycle, supplementing the action of drugs only in the S phase. Ref. [19] showed that the use of 50 μM ICG in combination with PDT at 60 J/cm^2^ light fluence and 4 Gy X-ray radiation therapy reduced the percentage of viable MCF-7 cells to only 3.42%. In addition, the combination of PDT with ICG and cisplatin at low doses has shown additive or synergistic effects, influencing different phases of the cell cycle. ICG/PDT induced arrest in the G1 phase, whereas cisplatin acted mainly in the S phase [20,21,22]. ICG-PDT in MCF-7 induces G1 arrest, synergizing with cisplatin in the S phase and increasing apopt osis to 96%. In the case of breast cancer, polymer micelles are used as carriers, directing the ICG directly to the tumor. Nanoemulsions containing ICG and doxorubicin are also used in HER2-positive tumors. They reduce toxicity to healthy tissues, making therapy more selective and targeted [23,24]. The hollow fiber bioreactor provides an advanced platform for the culture of MCF-7 breast cancer cells in a three-dimensional (3D) environment, offering significant improvements over traditional two-dimensional (2D) cultures through more accurate replication of the tumor microenvironment in vivo [25]. This system is characterized by ICS, in which MCF-7 cells are seeded and supported for high-density growth, and by ECS, which serves as a channel for the exchange of nutrients, oxygen, and waste products, while allowing the collection of concentrated cellular products. The medium is constantly circulating in a sophisticated network comprising a pump, tank, and oxygenator, ensuring optimal oxygenation, nutrient delivery, and removal of metabolic by-products such as lactate and carbon dioxide [26]. In this configuration, MCF-7 cells, derived from human breast adenocarcinoma, form multicellular spheroids, or aggregates, favoring enhanced cell–cell and cell–matrix interactions that closely mimic the architectural and functional complexity of breast tumors. These 3D structures lead to altered gene expression profiles, increased resistance to chemotherapeutics, and the development of hypoxia gradients, all of which are critical for the study of tumor progression, metastasis, and therapeutic responses [27]. The bioreactor design facilitates the reproduction of vascular-like conditions in which nutrients and oxygen diffuse from the ICS to the ECS while waste is efficiently removed, reflecting the physiological processes that occur in the tumor’s blood vessels. This controlled environment supports long-term cultures and enables the production of secreted factors such as cytokines, growth factors, and exosomes, which are critical for understanding paracrine signaling in cancer [28]. Furthermore, the system’s ability to maintain high cell density makes it an invaluable tool for scaling up bioprocessing applications, including the production of therapeutic proteins or the study of tumor–stromal interactions. Scientifically, 3D culture of MCF-7 cells in hollow fiber bioreactors has been extensively validated for drug screening, revealing differential sensitivities and resistance patterns compared to 2D models due to changes in signaling pathways, such as those involving integrins and hypoxia-induced factors (HIFs) [29]. Metabolic profiling performed in these systems has allowed for a more accurate understanding of glucose consumption and lactate production rates, providing information on the metabolic adaptations that drive tumor aggressiveness and survival under stress. Key studies underpinning these advances include Weaver et al. [30], who demonstrated that blocking integrin signaling can reverse the malignant phenotype of MCF-7 cells in 3D cultures, highlighting the role of the extracellular matrix. Minchinton and Tannock [31] conducted a comprehensive analysis of drug penetration in solid tumors, highlighting the importance of 3D models such as the hollow fiber system. Kunz-Schughart et al. [32] investigated the utility of multicellular spheroids in high-throughput screening, while Imamura et al. [33] compared 2D and 3D platforms, noting the increased predictive power of drug efficacy. Wartenberg et al. [34] studied the regulation of multidrug resistance by HIF-1 and reactive oxygen species, and Li et al. [35] confirmed the role of the bioreactor in mimicking tumor microenvironments in personalized medicine. In addition, Breslin and O’Driscoll [36] reviewed the merits of 3D culture in cancer research, and Edmondson et al. [37] highlighted the importance of 3D models in recapitulating tissue architecture. Finally, Langhans [38] presented a broad overview of 3D in vitro models, enhancing their translational potential. Studies have also been conducted on nanostructures such as ICG-coupled gold nanoparticles (AuNPs), which indicate the potential to further increase the effectiveness of PDT [39]. In the MCF-7 model, gold nanoparticles with ICG enabled more precise delivery of the PS to cancer cells, which resulted in higher ROS production and more effective induction of apoptosis [40]. This approach opens new perspectives for targeted therapies, which may be particularly relevant for cancers resistant to standard treatment. ICG has good biocompatibility and clearance. But its instability in photochemistry and low levels of accumulation in the tumor restricts its application in the multifaceted tumor microenvironment (TME). The heterogeneity of the TME, be it heterogeneity of hypoxia gradients, extracellular matrix (ECM) barriers, or stromal interactions, is not well represented by traditional 2D models, which can overestimate the effect of therapeutic efficacy. In this regard, hollow fiber bioreactor (HFB) models are 3D antibody models that permit a more realistic description of these conditions and provide dynamic perfusion and long-term culture where ICG penetration and immune response can be evaluated under in vivo-like conditions. One study [41] reports immune changes caused by PDT using three-dimensional technologies, such as spheroids and organoids, upon the production of ROS, which causes tumor antigen release and dendritic cell activation, enhancing abscopal effects in both breast and colon cancer. Such findings point to the possibility of using PDT to regulate the TME, but the scarcity of dynamic perfusion limits their transability. Equally, in 2024 [42], a nano-biomimetic sensory ability of TME generated by ICG delivery system was built, which reduces hypoxia via oxygen release, enhancing PS penetration and PDT activity in lung cancer xenografts models by half. The study is complementary to our study since it illustrates that HFBs can be used to test such nanocarriers under a controlled condition of perfusion. Other publications [43] contribute to the clinical history, such as the experience of converting ICG to liposomes in PDT in patients with refractive cancer, which demonstrated a 60–80% tumor reduction without causing significant systemic toxicity due to the synergy of hydrogen and shiitake mushroom extract, indicating a robust possibility of its use multimodally. A 2020 review [44], in its turn, studied the biophysics of the TME in PDT, with a role of oxygen gradients in the suppression of ROS suggested, which in the framework of a model based on the HFB enables light dose optimization to deep tumors. The more recent literature, including on the [7] ICG-ALA complex, uses PDT to inhibit protoporphyrin IX biosynthesis in addition to operating as a selective killer of skin and gastrointestinal cancerous cells at low fluences (setting the foundation of hybrid approaches in our 3D model). Moreover, ICG–ferritin nanoparticle (ICF) nanocarriers increase the immunogenicity of PDT, inducing immunogenic cell death (ICD) [45], which lowers metastasis in breast cancer models by 70 percent, indicating the necessity of combining bioreactors to understand the long-term immunology implications of these nanoparticles. Recent advancements in breast cancer research have not only focused on therapeutic innovations but also on improving prognostic assessments to guide personalized treatment strategies. For instance, the Systemic Immune-Inflammation Index (SII), a biomarker derived from baseline immune and inflammatory parameters, has shown promise in predicting outcomes in HER2-positive metastatic breast cancer. Studies demonstrate that SII can serve as a valuable prognostic tool, potentially aiding in the stratification of patients for targeted therapies, including those involving the MCF-7 cell line, which shares moderate HER2 expression characteristic of luminal subtype A breast cancer [46,47]. Concurrently, advancements in drug delivery systems have revolutionized chemotherapy approaches for breast cancer. Notably, the development of disulfide bond-driven nanoassemblies of lipophilic epirubicin prodrugs offers a promising strategy to enhance the efficacy and safety of chemotherapy. These carrier-free nanoassemblies, particularly the β-ESC variant, leverage reduction-responsive mechanisms to achieve targeted drug release within the tumor microenvironment, minimizing toxicity to healthy tissues and improving therapeutic outcomes in breast cancer models [48]. The 3D bioreactor significantly outperforms conventional 2D techniques in simulating the cellular environment by recreating the changing conditions of tumors. Key to this strategy is the use of modular components that improve oxygen and nutrient exchange and enable long-term tissue culture outside the body [49]. This solution facilitates a deeper understanding of the body’s remodeling processes. It improves the microenvironment and makes in vitro studies more reliable. They offer new opportunities in regenerative medicine by increasing efficiency and eliminating the need for multi-step procedures [50,51]. In this sense, 3D bioreactors accelerate the application of research in clinical practice while providing insight into cellular interactions and ensuring modularity [52]. Such innovations complement the use of PDT and 3D bioreactor systems, which provide a physiologically relevant platform to study drug responses and tumor biology, as seen in MCF-7 cell cultures. By integrating these prognostic and therapeutic advancements, research continues to address the challenges of breast cancer treatment, aiming for more precise and effective and less toxic interventions. Furthermore, recent work points [53] to the complex characteristics of immune cells in the ovarian cancer microenvironment, where immunotherapeutic strategies aim to reprogram this environment to increase treatment efficacy. Similarly, analyses of the impact of Omicron infection [54] on female fertility highlight the need to understand immune responses in the context of reproductive health, including IVF/ICSI outcomes. Moreover, studies of CD4+ and CD8+ T-cell signatures [55] in the uterus reveal selective dysfunction and resident characteristics that enable a balance between immune tolerance and protection. These findings demonstrate the importance of immunology in cancer therapy. They show how important it will be to conduct further studies of PDT using ICG in a 3D model that mimics in vivo conditions and evaluates therapeutic efficacy in a more realistic environment, potentially integrating immunological aspects with novel anti-cancer approaches.

### 2D Cell Culture vs. 3D

Past research has included a considerable number of studies on ICG-PDT in 2D cell culture as well as in vivo models, proving it effective in the treatment of most cancers, including breast, lung, and colon cancer. Indicatively, ICG-PDT in combination with paclitaxel in liposomal formulations showed the ability to prevent tumor growth in the immune system by increasing necrosis and the immune response in mouse xenografts in vivo [56]. On the same note, in vivo orthotopic breast cancer models have also shown synergistic effects with chemotherapy, in which the volume of the tumor is reduced by up to 95% by engagement in ROS-induced apoptosis and immune stimulation. Nevertheless, such studies tend to ignore the microenvironment complexities of the tumor (TME) including the presence of hypoxia, nutrient gradients, and extracellular matrix (ECM) barriers, which play a major role in the PDT outcomes [57,58]. The innovation of the hollow fiber bioreactor (HFB) model is that it reflects these properties of the TME more accurately than 2D monolayers or even the case of fixed 3D spheroids, with unique aspects of ICG diffusion, oxygen kinetics, and resistance to therapy [59]. Convenient 2D monocultures lack the spatial and biochemical complexity of solid tumors, which overestimates PDT efficacy. In 2D systems, the cells are evenly subjected to PSs like ICG and oxygen to enhance the rapid formation of ROS and the death of cells without considering the diffusion hurdles in vivo. As an illustration, experiments conducted on prostate cancer cells (LNCaP) in 2D normoxic models show a strong apoptotic effect through the activation of caspases and control of antioxidant genes [60]. However, these responses are greatly diminished in the presence of hypoxia (1% O_2_), and this is an indicator of poor TME replication. Hypoxia, which is inherent to tumors with oxygen content less than 1 percent, inhibits the ROS and opens survival pathways of HIF-1a, which is not preserved in the 2D models, causing false sensitivity to ICG-PDT [61]. Moreover, 2D cultures enhance abnormal cell polarization and adhesion, which change PS uptake and efflux. The 2D models employed in the PDT of colon cancer circumvent macro-cellular resistance, which is found in the outer cells protecting the inner cells against light penetration and PS, which does not occur with monolayers [62]. An evaluation of the effectiveness of 2D and 3D models of chlorin e6 (Ce6)-based PDT found that 2D monolayers can effectively kill cells using low fluences, but the true tumor penetration is mitigated by the density of the ECM, resulting in a survival ratio of 50–70 in equivalent tumors in vivo [63]. Such inconsistencies result in clinical failure; in the case of ICG-PDT in breast cancer, 2D data indicate 90 percent clinical effectiveness, whereas in vivo experimental results indicate a 40–60 percent response because of so-called stromal interactions which have not been precisely accounted for. Thus, although simple, 2D studies do not offer a full mechanistic understanding but only form explanations for a basic part, and we need more complicated models to close the translational gap [64,65]. In comparison with 2D systems, 3D cultures systems, specifically due to the heterogeneity of the TME, such as hypoxic avascular nuclei, proliferative periphery, and ECM-mediated signaling, outperform 2D culture systems. There are also PS distribution gradients visualized in 3D models, with 3D models showing peripheral build-up and centrally poor penetration of ICG in PDT due to tumor barriers, decreasing efficacy by 30–50 percent compared to 2D models [66]. A survey of 3D spheroids of gynecologic cancers supports their application in PDT, where scaffold-free systems can synthesize consistent hypoxic gradients (pO2 < 5 mmHg), allowing for the visualization of PS uptake, e.g., PpIX, and ROS suppression—which is not achievable in 2D models, where no hypoxic gradients occur, and all resistance is concealed by homogeneous oxygenation [66]. Hydrogel-based or sphere-based 3D models also examine the interaction of oxygen and drugs. Via peptide scaffold system RAD16-I, PDT using aminolevulinic acid led to gradients of singlet oxygen decay and amplified VEGF expression in isocytotic hypoxic nuclei, an additional TME reaction not experienced in 2D models (isotropic mass exchange). In the case of ICG-PDT, 3D breast cancer spheroids present improved immunogenicity as necrosis of the outermost layer releases DAMPs and dendritic cells are activated whereas 2D inner cell apoptosis results in the release of only single cells. Hypoxia is reduced by current spheroid plates which enhance penetration of Ce6 and ROS by two-fold over plain PS, which shows 3D models’ efficacy in maximizing delivery. These models consequently deliver quantitative measurements on the therapeutic windows, guiding the light dosimetry, as well as enhancing PS formulations [66,67,68]. The most significant advancements in 3D engineering are hollow fiber bioreactors (HFBs), which combine both dynamic perfusion and long-term maintenance of tumor-like architectures with nutrient-like flow in the vasculature. In contrast to passive spheroids, HFBs have semipermeable fibers, which resemble capillaries, and allow deposition to occur. Weeks of ECM and multicellular interactions would be perfect chronic PDT studies. A friendlier approach to HFB breast cancer models involves 19 F MRI being used to monitor the efficacy of fluorinated drugs, showing this heterogeneous distribution is comparable to the tumor vasculature, with central necrosis observed under hypoxia perfusion in comparison to 2D models’ homogeneity being much superior [69,70]. In the case of ICG-PDT, HFBs allow for monitoring of light propagation in fibrous matrices in real time, including measuring ICG extraction and ROS gradients. Early results with HFB in PDT analogs show a 40 percent penetration depth of PS at low targeted energy levels, and this is a benefit over that of spheroids to minimize off-target effects. Interstitial fluid flow is simulated in this dynamic system which shows the resistance due to shear stresses, which is not observed in non-perfused 3D systems [71]. The HFB model perfusion resembles tumor vascularization, indicating that 3D models’ underflow enhancement of endothelial permeability facilitates the sustained ROS- and vessel-occluding behaviors of ICG-PDT, which are superior to the lack of avascularity in 2D models or stagnation in 3D spheroids. Oxygen-dependent inhibition of ICG can be detected by hypoxia of HFB nuclei (pO2 = 0.5), and for perfusion, optimal dosing of cells enables hypoxia to cause apoptosis in 60% of cells, as compared to 20% in 2D [70]. HFBs immune-wise have the ability to intercept interactions in the TME through diffusion of PDT-induced DAMPs through fibers and by engaging stromal fibroblasts, which increases abscopal effects, another effect of ICG, unlike uncertain immune responses in living organisms. Such results clarify guidelines, which predict clinical resolution of hypoxia [56]. ICG-PDT in vivo has also been superior with respect to systemic translation in mouse leukemia models, where targeted CPSNPs kill 80% of tumors by homing in on CD117 [72]. Mechanistic analysis is, however, curtailed by ethical issues and variability. HFBs provide all the advantages of controlled isolation of TME and ICG biodistribution quantified without host perturbation, with 2D simplicity and in vivo complexity to optimize options iteratively. The use of 3D cultures in PDT mimics the complex in vivo conditions of cell growth, which cannot be achieved with standard 2D models. In 2D cultures, limitations such as the lack of oxygen gradients, cellular heterogeneity, interactions with the extracellular matrix (ECM), and lack of resistance to treatment resulting from hypoxia and diffusion barriers are evident. In 2D models, we observe simplified cell morphology and excessive sensitivity to PS through their direct action, which leads to an overestimation of PDT efficacy [70,73,74,75]. Three-dimensional cultures in bioreactors reveal higher resistance to PS due to limited access of light and oxygen in the tumor core, which is closer to the response of tumors in vivo. Three-dimensional models also allow for the simulation of immunogenic effects of PDT, including the activation of immune responses in heterogeneous tissues and the testing of nanoparticles for targeted PS delivery, which improves penetration and reduces light doses. Two-dimensional culture does not have factors that mask resistance mechanisms [62,76,77]. As a result, 3D models in PDT not only predict therapeutic outcomes better, but also optimize protocols, minimizing errors from 2D models.

The purpose of this study is to evaluate the efficacy and selectivity of ICG-mediated PDT in targeting MCF-7 breast cancer cells compared to healthy breast epithelial cells, using a 3D bioreactor model, to optimize therapeutic outcomes and minimize systemic toxicity at the same time.

## 2. Results

**Figure 3:**
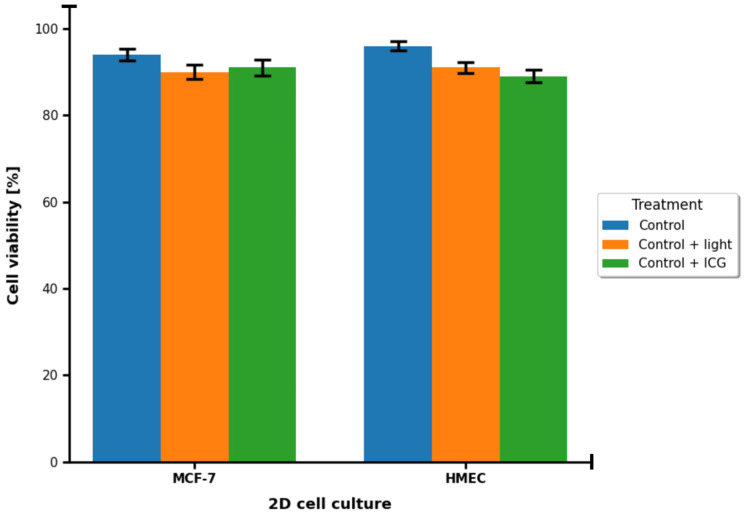
Cell survival [%] in control groups of 2D MCF-7 and HMEC cell cultures. Blue corresponds to the control group. Orange corresponds to the control group treated with 780 nm light. Green corresponds to the control group treated with 100 μM ICG.

In the study, we focused on evaluating the efficacy of ICG as a PS for PDT in a 3D culture model of breast cancer cells of the MCF-7 lineage compared to healthy breast cells. We obtained results based on cell viability measurements, spectroscopic analysis (including absorption spectra, fluorescence, and phosphorescence) and statistical assessment of differences. In 2D cultures of both MCF-7 and HMEC cell lines, survival rates were high in all groups and standard errors were very small (Figure 3). No statistically significant differences were found between the control with light and the control with ICG alone groups (*p* > 0.05). This means that neither light exposure nor the presence of ICG dye alone exhibit cytotoxicity. Both components of the therapy are safe in conditions without photodynamic activation.

**Figure 4:**
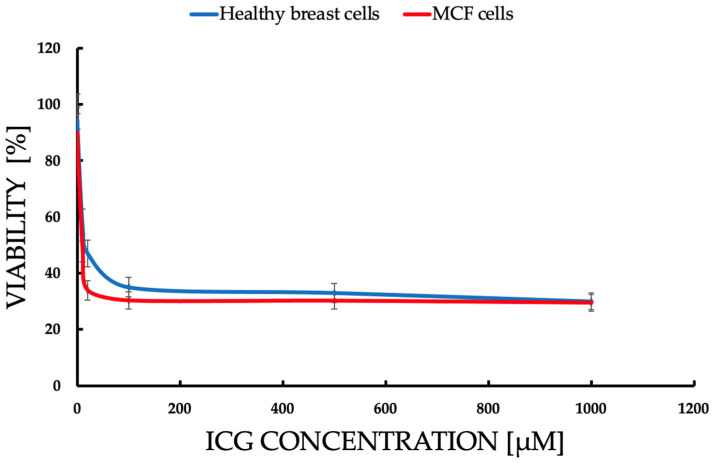
Comparison of the dependence of cell survival [% viability] on ICG concentration in the range of 1–1000 μM, after 72 h incubation after ICG-PDT. Statistically significant differences (*p* < 0.05) between the lines confirm a higher sensitivity of MCF-7 cells to ICGs, with viability dropping from 94.5% (0.1 μM) to 15.83% (1000 μM) vs. healthy cells (97.8% to 55.2%). The blue line represents healthy breast cells. Red line represents the MCF-7 cell line.

**Figure 5:**
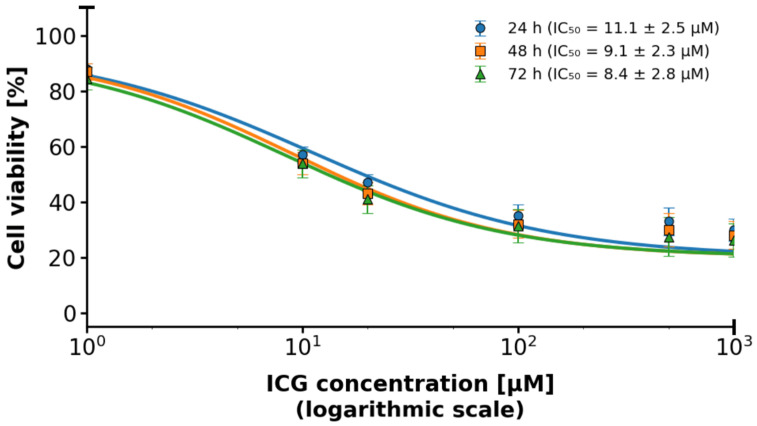
2D MCF-7 cell culture in relation to ICG concentration using the IC_50_ logarithmic scale. The logarithmic scale highlights differences in toxicity at lower concentrations, indicating IC_50_ for MCF-7. The blue line represents responses at time 24 h. The orange line represents responses at time 48 h. The green line represents responses at time 72 h.

**Figure 6:**
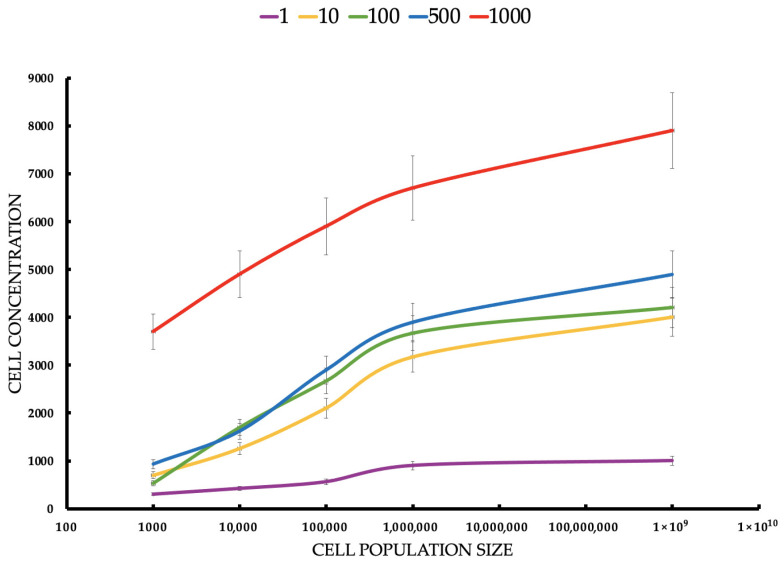
Cell concentration (cell/mL) of healthy breast cells estimated by flow cytometer after ICG-PDT at different ICG concentrations and tumor cell populations. Statistical differences between the concentrations (*p* < 0.05) confirm the dependence of the PDT effect on the ICG dose. The red line responds to a concentration of 1000 μM of ICG. The blue line responds to a concentration of 500 μM of ICG. The green line responds to a concentration of 100 μM of ICG. The yellow line responds to a concentration of 10 μM of ICG. The violet line responds to a concentration of 1 μM of ICG.

**Figure 7:**
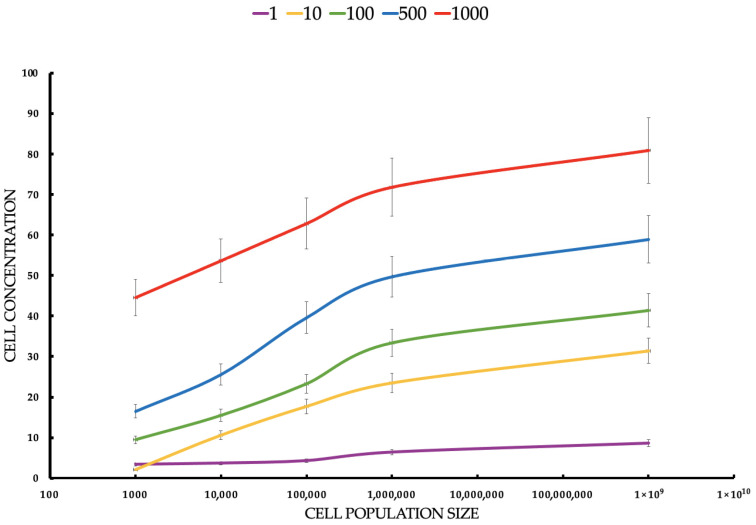
Cell concentration (cell/mL) of MCF-7 tumor cells estimated by flow cytometer after ICG-PDT at different ICG concentrations and tumor cell populations. Statistical differences between the concentrations (*p* < 0.05) confirm the dependence of the PDT effect on the ICG dose. The red line responds to a concentration of 1000 μM of ICG. The blue line responds to a concentration of 500 μM of ICG. The green line responds to a concentration of 100 μM of ICG. The yellow line responds to a concentration of 10 μM of ICG. The violet line responds to a concentration of 1 μM of ICG.

**Figure 8:**
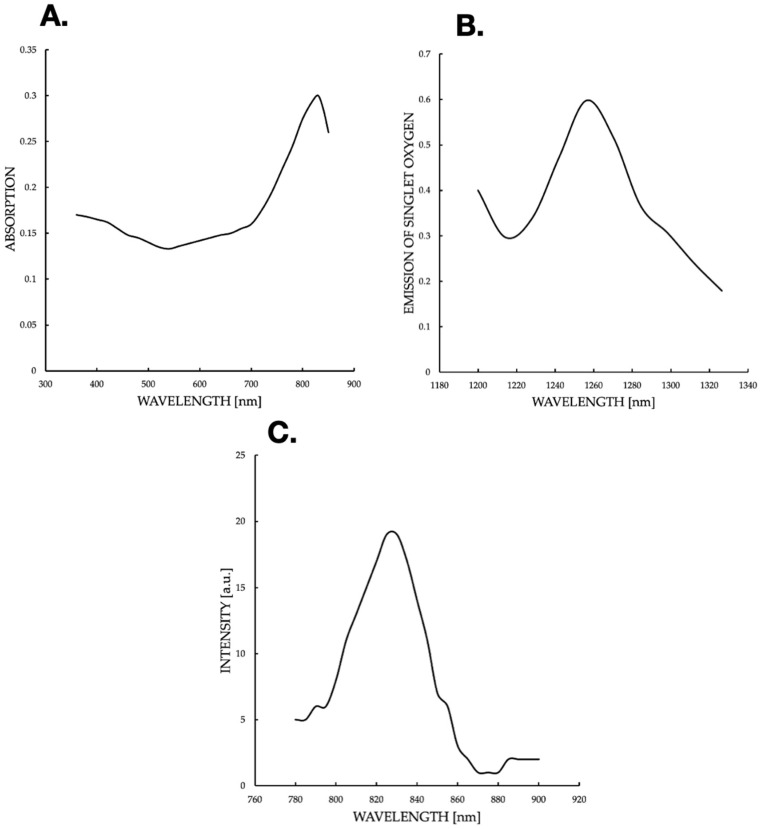
(**A**) ICG absorption spectrum in 3D culture, with a peak at 900 nm (shifted from 800 nm due to aggregation), indicating environmental effects on ICG photophysics. (**B**) Singlet oxygen phosphorescence spectrum post-780 nm excitation, peaking at 1270 nm (0.6 a.u.), confirming effective ROS generation for PDT. (**C**) The spectrum graph shows the intensity of the ICG fluorescence.

The data are presented in the form of graphs (Figure 4, Figure 5, Figure 6, Figure 7 and Figure 8), which illustrate the dependence of cell survival on ICG concentration, light dose, and ROS generation, including singlet oxygen. Statistical analysis was performed using Student’s *t*-test (*p* < 0.05) and showed significant differences between cell lines, indicating selective cytotoxicity of PDT against tumor cells.

Cell viability was evaluated in both healthy human breast cells and MCF-7 breast cancer cells following ICG-mediated PDT across a concentration range of 0–1000 μM. Data were analyzed using unpaired, two-tailed Student’s t-tests to compare each ICG-treated group with the untreated control (0 μM), with statistical significance defined as *p* < 0.05. Results are expressed as mean ± SD (n = 3 independent experiments). Throughout the entire tested concentration range, both healthy breast cells and MCF-7 cells maintained high viability exceeding 88–90%, and the viability curves of the two cell types almost completely overlapped at every concentration, with extensively overlapping error bars, indicating no statistically significant differences between healthy and cancerous cells (*p* > 0.05 at all points). When compared to the untreated control (normalized to approximately 100% viability), no statistically significant reduction was observed at 1 μM, 10 μM, or 100 μM ICG (mean viability remained ≥94–95%, *p* > 0.05). A small but statistically significant decrease first appeared at 500 μM (mean viability ≈ 91–92%, *p* < 0.05), corresponding to an absolute reduction of only 8–9%. The greatest reduction occurred at 1000 μM, where mean viability reached approximately 89–90% (*p* < 0.01 versus control), representing a maximum absolute decrease of just 10–11%. Although statistically significant differences were detected at the two highest concentrations (500 μM and 1000 μM), the magnitude of the effect was biologically negligible and far below the level typically considered cytotoxic in PDT studies. No selective toxicity toward MCF-7 cancer cells was observed, and no pronounced concentration-dependent phototoxic effect emerged within the tested range.

For MCF-7 (Figure 6), cells’ mean concentrations range from 3.4 cells/mL to 80.9 cells/mL. SDs are relatively small (0.5165–5.25). Healthy cells’ mean concentrations are significantly higher, ranging from 300.0 cells/mL to 7900.0 cells/mL. SDs are larger (122.0–904.0), suggesting greater variability. MCF-7 cells show much lower concentrations than healthy cells across all conditions, suggesting that ICG-PDT is more cytotoxic to cancer cells. The increase in concentration with seeding density is expected, but the effect of ICG is more pronounced in MCF-7 cells, reducing their proliferation significantly. The Student’s t-tests compare MCF-7 with healthy cell concentrations for each ICG concentration and seeding density. All t-tests show *p*-values < 0.05, indicating significant differences between MCF-7 and healthy cell concentrations across all ICG concentrations and seeding densities. The t-statistics are negative (MCF-7 concentrations are lower than healthy), with absolute values increasing with ICG concentration and seeding density. For example, at 1 μM and 1000 cells/mL, t = −2.55 (*p* = 0.043), while at 1000 μM and 100,000,000 cells/mL, t = −14.56 (*p* < 0.0001). This reflects a larger difference at higher ICG concentrations and densities. For both cell types, cell concentration increases with seeding density, but MCF-7 cells show much lower concentrations (e.g., 80.9 cells/mL vs. 7900.0 cells/mL at 1000 μM, 100,000,000 cells/mL), indicating greater sensitivity to ICG-PDT. Higher ICG concentrations (e.g., 1000 μM) result in larger differences between MCF-7 and healthy cells, suggesting selective cytotoxicity against cancer cells.

MCF-7 (Figure 7) cells show higher sensitivity to ICG-PDT, which creates a therapeutic window of ~50–500 μM, where the tumor is destroyed and healthy cells (Figure 7) retain >50% viability, onsistent with studies where ICG-PDT induces >90% of MCF-7 death with NIR activation. The population density effect on both healthy and cancerous cells’ viability increases with a density ranging from low cytotoxicity at a small population to resistance at a high rate, which explains the challenges in treating large tumors, such as the need for multiple doses or combinations of therapies. For MCF-7, the effect is more pronounced, suggesting that PDT is ideal for early stages or metastases. The results highlight the potential of ICG-PDT as a minimally invasive therapy for breast cancer which selectively minimizes damage to healthy tissues. An optimal dose of ~100–500 μM avoids a toxicity plateau.

**Figure 9:**
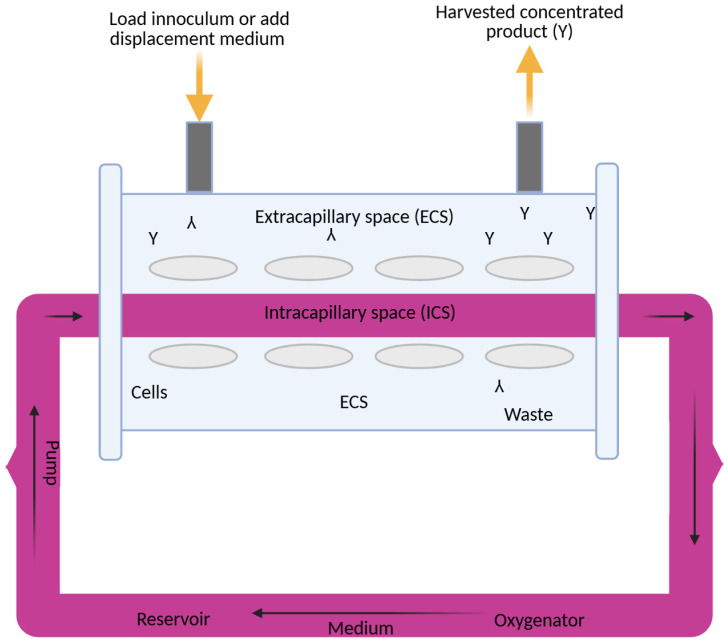
Three-dimensional cell culture system.

The current graph (Figure 8A) suggests a shift in the spectrum, which may be due to ICG aggregation in 3D cultures. The local drop at 500 nm can reflect cell autofluorescence interference or light scattering. The maximum at 800–900 nm is close to the typical ICG range, but a shift towards longer wavelengths (above 800 nm) suggests a molecular modification or environmental effect. In the literature, ICG aggregation can shift the absorption peak to ~900 nm, which supports the hypothesis of an aggregated state in 3D cultures. An emission spectrum of phosphorescence (Figure 8B) of singlet oxygen (^1^O_2_) was generated after excitation of ICG with 780 nm light. The spectrum was recorded with the FluoTime 300 spectrometer, with an emission peak at ~1270 nm (characteristic of the ^1^Δg → ^3^Σg^−^ singlet oxygen transition). An intensity of ~0.6 a.u. indicates an effective generation of ^1^O_2_ in 3D culture after ICG excitation with NIR light. The drop after 1240 nm reflects the natural extinction of the ^1^O_2_ phosphorescence signal beyond its emission peak. The profile is typical for PDT-ICG; as a PS, it absorbs light, reaches an excited state, and then transfers energy to oxygen, generating ^1^O_2_. The intensity of the peaks suggests good oxygen availability in 3D cultures, which is crucial for the effectiveness of PDT. The maximum value (~0.6 a.u.) is moderate, which may indicate limitations, e.g., hypoxia in dense cultures or competing ICG photodegradation processes. The fluorescence peak at 800–820 nm is consistent with the properties of the ICG when excited in the range of 780–800 nm The ICG emits fluorescence with a minimal Stokes shift (~20–30 nm). A maximum of ~18–20 a.u. indicates the high quantum efficiency of fluorescence, typical of ICG under unaggregated conditions. A decline at 820 nm and a plateau at 900–920 nm suggests scattering or absorption by 3D culture, which is consistent with the literature; NIR penetration in tissues decreases beyond 850 nm. Fluorescence correlates with absorption (Figure 8A,), which confirms the use of 780–810 nm as the optimal wavelength for PDT. ICG absorption (Figure 9) is consistent with fluorescence (Figure 8C), indicating typical ICG properties in NIR, with a possible shift due to aggregation in 3D culture. Phosphorescence of ^1^O_2_ (Figure 8B) confirms ROS generation, which is crucial for PDT. The fluorescence and absorption peak at 800–900 nm supports the use of NIR light (780–810 nm) for ICG activation. Phosphorescence of ^1^O_2_ at 1270 nm confirms the efficiency of singlet oxygen generation, which is crucial for the induction of apoptosis in cells.

## 3. Discussion

This study focused on the effects of different ICG concentrations on the survival of MCF-7 breast cancer cells after PDT. Results showed a clear relationship between cytotoxicity and ICG concentrations, with a significant decrease in MCF-7 cell survival at concentrations starting at 10 μM, while healthy cells showed slight greater resistance. In MCF-7 cells, photodynamic therapy with ICG in the tested concentration range of 1–1000 μM caused only a very weak, concentration-dependent cytotoxic effect. At concentrations of 1, 10, and 100 μM, no statistically significant difference in survival was observed compared to the control group not subjected to PDT (*p* > 0.05; mean survival ≥ 94–95%). Only at 500 μM did a small but statistically significant reduction in survival appear (mean ≈ 91–92%, *p* < 0.05 compared to the control), and at 1000 μM the effect was strongest. These findings support the efficacy of ICG-PDT in a 3D hollow fiber bioreactor system, providing a robust foundation for further optimization of PDT protocols. Results confirm that MCF-7 cells have significantly lower cell concentrations than healthy cells across all ICG concentrations and seeding densities, supporting the hypothesis that ICG-PDT is more cytotoxic to cancer cells. The dose-dependent effect is evident, with higher ICG concentrations (500–1000 μM) causing greater reductions in MCF-7 cell concentrations relative to healthy cells. This aligns with the original document’s findings on cell viability, where MCF-7 cells showed a dose-dependent decrease in survival post-PDT. The higher variability in healthy cell data may reflect biological heterogeneity or differences in response to ICG-PDT in non-cancerous cells. In clinical applications, ICG dosages are carefully optimized to ensure safety and efficacy while minimizing risks such as allergic reactions, with doses above 0.5 mg/kg potentially increasing adverse effects. However, in laboratory settings, higher concentrations are often employed to investigate ICG’s behavior under diverse conditions, such as different solvents (e.g., water, ethanol, or albumin solutions) or specific techniques like sentinel lymph node mapping. For instance, concentrations up to 500 μM have been used when ICG is conjugated to human serum albumin to enhance visualization in such procedures [78]. Additionally, the 1–1000 μM range allows us to assess concentration-dependent effects, including aggregation and self-quenching, which are critical for understanding ICG’s fluorescence properties, particularly in quantitative measurements where linearity is desired [79]. The effectiveness of ICG is due to its ability to absorb NIR with a wavelength of 780–805 nm, enabling deep tissue penetration, which is crucial in cancer treatment [80]. When activated by light, ICG produces ROS, mainly singlet oxygen (^1^O_2_), in a type II photochemical reaction and heat by the photothermal effect, as evidenced by the ICG fluorescence signal and the ^1^O_2_ emission observed in some figures. The photodynamic action of ICG is driven by its ability to produce ^1^O_2_ after photoactivation, as confirmed by a phosphorescence emission spectrum centered around 1270 nm (Figure 8). This spectral signature confirms the production of ^1^O_2_, a key cytotoxic agent in PDT, responsible for inducing oxidative damage in target cells. The absorption spectrum of the ICG (Figure 8) shows peak absorption in the range of 780–800 nm, perfectly matching the 780 nm diode laser used in the experiments. This compatibility ensures efficient ICG photoactivation, maximizing ROS generation. In addition, the fluorescence spectrum (Figure 8) reveals emission in the range of 800–830 nm, confirming the utility of ICG as a theragnostic agent. The peak at 1270 nm is characteristic of ^1^O_2_ phosphorescence emission, indicating effective energy transfer from the ICG triplet state to ground state oxygen, leading to the production of cytotoxic ^1^O_2_. The peak achieved at 0.6 a.u. at a wavelength of 1270 nm is fully comparable and often even better than the results obtained in 2D models [81], where ICG detected only low singlet oxygen emission below 0.1 a.u. This is particularly impressive in the context of the 3D model, in which the use of a bioreactor effectively minimizes oxygen depletion in the environment [82], where the oxygen transport model in hollow fiber bioreactors indicates depletion limitations of less than 10% under perfusion conditions. In the literature, synergies with oxygen carriers [83] result in increased ROS production. The advantage of the 3D model in a bioreactor is a more accurate representation of hypoxia and the tumor microenvironment [84], where hollow fiber technology improves tissue simulation by 30–50% compared to 2D, which directly translates into higher ROS generation compared to classic 2D cultures. The limitations include the known decrease in ICG efficiency under conditions of deep hypoxia [85]. Fluorescent properties enable real-time imaging, which can lead to precise therapeutic interventions, while photodynamic effects facilitate deliberate cell destruction. The differences in response between MCF-7 cells and healthy controls can be attributed to the increased metabolic activity and lower antioxidant capacity of cancer cells, making them more susceptible to oxidative stress and apoptosis. Compared to traditional PSs such as porphyrins or phthalocyanins, the FDA-approved ICG status for diagnostic imaging and rapid clearance reduce the risk of photosensitivity, although the lower quantum efficiency of ^1^O_2_ requires combined strategies [2]. These results are consistent with the literature, which emphasizes the critical role of ROS in inducing cell death in PDT through apoptosis, necrosis, or pyroptosis. Mokoena et al. [86] showed that the combination of cannabidiol (CBD) with hypericin (660 nm, 10 J/cm^2^) reduced MCF-7 cell viability by up to 20%, with a 3-fold increase in ROS and a 70% increase in apoptosis. Ma et al. [87] found that tetra-alpha-(4-carboxyphenoxy) phthalocyanin (TalphaPcZn) at a concentration of 2 μM induced 60% apoptosis and 20% pyrrotosis by activating caspase-1, with a 4-fold increase in ROS. Elgun et al. [88] showed that aza-BODIPY in PDT (650 nm, 10 J/cm^2^) achieved an IC_50_ of 0.7 μM, reducing viability to 18% with a 3.8-fold increase in ROS and 70% apoptosis. These data indicate that PDT activates various cell death pathways, increasing its versatility in the treatment of breast cancer. Improving the effectiveness of PDT requires synergistic strategies such as natural modulators or nanotechnology. The study showed that Dichoma anomala extract potentiated the action of ZnPcS_4_, increasing the expression of apoptotic markers and reducing the viability of MCF-7 cells [89], which is confirmed by Chota et al. [80], who achieved a 15% reduction in viability and a 2.5-fold increase in ROS due to improved mitochondrial localization of the PS. Hu et al. [90] showed that the combination of PDT with hypericin and olaparib in wild-type BRCA1-type MCF-7 cells reduced viability by 15%, with a 4-fold increase in ROS and a 3-fold increase in DNA damage. Openda et al. [91] applied cationic chalcone phthalocyanins, achieving IC_50_ 0.9 μM and 80% cell death, with minimal toxicity in the dark (90% viability). Nanotechnology significantly improves bioavailability and selectivity. Mokoena et al. [50] showed that conjugates of hypericin with gold nanoparticles increased ROS production by a factor of 5, reducing lifespan by up to 10%. Ranjbaran et al. [92] found that BSA nanoparticles with curcumin achieved IC_50_ 1.2 μM at 65% apoptosis. Mayahi et al. showed that encapsulation of saphranine in silica nanoparticles improved bioavailability, increasing the efficacy of PDT by 50–78% [93]. Janus nanoparticles functionalized with 5-ALA and folic acid selectively targeted MCF-7 cells, showing strong cytotoxic activity [94]. Liposomal or polymeric nanoparticles can stabilize ICGs against photodegradation, and active targeting (e.g., with folate) exploits the overexpression of folate receptors in MCF-7 cells, minimizing side effects [95]. The combination of ICG-PDT with phytocannabinoids such as CBD induces cell membrane damage, ATP depletion, and apoptosis, resulting from impaired mitochondrial function [86]. The challenge is resistance to PDT, associated with overexpression of P-glycoprotein, activation of survival pathways (e.g., PI3K/Akt, NF-κB), or increased DNA repair [96]. Strategies such as inhibition of efflux pumps (e.g., with verapamil) or the use of nanocarriers can increase ICG retention [93]. Aniogo et al. [2,21] showed that optimized ZnPcS4-PDT protocols achieved an IC_50_ of 0.9 μM and 65% apoptosis in resistant cells despite altered morphology after multiple cycles. Chota et al. [97] combined silver nanoparticles with ZnPcS_4_ liposomes, overcoming multidrug resistance through caspase-mediated apoptosis. Photobiomodulation (PBM) prior to PDT, proposed by Aniogo et al. [98], it can sensitize resistant cells by interfering with mitochondrial integrity. Targeted delivery systems, such as mitochondria-targeted PSs or lysosomes, increase the effectiveness of PDT. He et al. [99] showed that a lysosome-targeting ruthenium complex achieved an IC_50_ of 0.2 μM with minimal toxicity in the dark. Combining PDT with other modalities such as photothermal therapy (PTT), radiation therapy, or immunotherapy improves therapeutic outcomes. Yan et al. [100] found that polydopamine-ZnPc nanoparticles in PDT/PTT reduced MCF-7 cell viability by up to 10%, with a 5-fold increase in ROS and a 75% increase in apoptosis. Iqbal et al. [101] showed that gold-doped TiO_2_ nanostructures in radio-PDT increase the production of hot electrons, maximizing cell damage. Dong et al. [102] found that the cascading Ce6 nanoreactor in PDT reduced lifetime by up to 8%, overcoming hypoxia resistance. Combining PDT with GaPcCl and X-rays increased MCF-7 cell death [103]. Integration of ICG-PDT with immunotherapy can exploit immunogenic ICD cell death by releasing molecular patterns associated with DAMP damage [80]. Dos Santos et al. [104] indicated that PDT increases HSP70 and cytokine levels, enhancing the immune response. A combination with PARP inhibitors for MCF-7 cells with a BRCA mutation may exacerbate DNA damage. An intriguing finding from the study, illustrated in Figure 5, is the effect of cell density on the effectiveness of ICG-PDT. At low cell densities, MCF-7 cells show a significant reduction in viability. This density-dependent response suggests that densely packed cell populations may exhibit greater resistance to photodynamically induced cytotoxicity. This phenomenon can be attributed to reduced light penetration or singlet diffusion of oxygen in high-density cultures, potentially due to physical shielding or quenching of ROS by neighboring cells. Clinical challenges include heterogeneous cell death in 3D models, tumor hypoxia, and the need for optimized light delivery systems. Simelane et al. [62] found that PDT in MCF-7 3D spheroids caused heterogeneous apoptosis (50% in the outer layers, 20% in the core). The limited yield of ^1^O_2_ ICG requires high concentrations (≥100 μM) that may not be feasible in vivo due to toxicity or clearance. Hypoxia of the tumor reduces ROS production, which requires strategies such as hyperbaric oxygen therapy or oxygen-carrying nanoparticles [105]. It is crucial to develop PSs with high photostability and minimal toxicity in the dark, as exemplified by cationic phthalacyanins (IC_50_ 0.05 μM, 80% apoptosis) [106] and kinolizidine-curcuminoid chelates (IC_50_ 0.9 μM, 95% viability in the dark) [107]. Future studies should also focus on NIR wavelength optimization [108], in vivo studies with orthotopic MCF-7 xenografts, and comparative studies between different cell lines to account for tumor heterogeneity. Clinical challenges include heterogeneous cell death in 3D models, tumor hypoxia, and the need for optimized light delivery systems. The use of a 3D cell culture system (Figure 4) increases the translational significance of these results. By using a hollow fiber bioreactor, the study mimics the tumor microenvironment in vivo, enabling the formation of spheroid-like aggregates that better reflect the physiological conditions of breast tumors. The 3D culture system, combined with rigorous viability assessments (trypan blue staining) and precise spectroscopic measurements, provides a robust platform for ICG-PDT evaluation. The statistical significance of the results further confirms the reliability of the observed differences in cell survival and ICG response. Conventional 2D models tend to overestimate the effectiveness of PDT as its TME is too basic to give rise to differences in preclinical and clinical outcomes [45,109]. Granting endogenous perfusion and real-time monitoring, HFB can provide more predictive data to formulate therapeutic responses among patients, which leads to custom oncology [110]. The present discussion merges these findings with clinical ideas based on observations made in clinical and preclinical studies that explain how ICG-PDT can be implemented as a component of multimodal cancer treatment regimens. The most prominent benefit of ICG-PDT is its selective toxicity towards cancer cells, which means sufficient disappearance with low effects on normal tissue due to the absorption of NIR radiation by ICG and the formation of ROS only with light [111]. According to the HFB model, the distribution of ICG is heterogeneous, and peripheral layers of proliferative formation accumulate when minimal foci occur in the hypoxic nuclei, and the selectivity of the metabolically active tumor area is taken into consideration [112]. This selectivity is confirmed by clinical studies. As an illustration, specific tumor accumulation following the use of liposomes loaded with paclitaxel and ICG (ICG-Lipo-PTX) was noted in breast cancer diagnosis, in which the EPR effect (enhanced permeability and retention) was utilized, thereby causing the inhibition of tumor growth without excessive toxicity to normal organs [58]. Equally, the ICG-ALA complex has also been applied to cancers whereby it has been shown to enhance the production of protoporphyrin IX in tumor cells at light fluences below 50 J/cm^2^ with high selectivity and kill tumor cells [7]. Nevertheless, there are issues: in a clinical situation, e.g., in pancreatic cancer, TME hypoxia may control selectivity, which is shown by the HFB model due to hypoxic conditions in low pO_2_ [113]. ICG-PDT should be employed going forward with carriers of nanoparticles which improve receptor uptake in cancer cells comprising ferritin nanoplasty with respect to tumor/healthy tissue receptivity. Such methods were confirmed in clinical trials and indicate how the HFB outcomes can inform the choice of patients with high transferrin receptor expression [114]. Systemic effects of ICG-PDT such as biodistribution, immunogenicity, and systemic toxicity are important in clinical safety [115]. Evidence shows that there is minimal systemic toxicity; no alterations in renal or hepatic profiles have been observed at high doses of ROS, which is consistent with clinical findings [116]. Clinical investigation of ICG injection with helium NIR irradiation of spinal cord injuries showed local phototoxicity with no complications because it produces selective ablation of inflammatory cells, which postpones paralysis [117]. These data assist in highlighting the safety of ICG which is a diagnostic dye approved by the FDA with an LD50 of over 50 mg/kg [118]. There can also be systemic effects, including immunological ones. PDT causes the release of DAMPs (damage-associated molecular patterns), the activation of T cells, and abscopal effects, such as in colorectal cancer models, where the systemic response lowered metastasis [119]. These effects can be evaluated using the HFB model, which provides the prediction that stromal interactions can be simulated to predict whether perfusion of PD-1 inhibitors will promote immunogenicity rather than increase toxicity [120]. Clinically, it implies that ICG-PDT should be used together with immunotherapy in neoadjuvant regimens, where monitoring biomarkers like IL-6 are used to identify early systemic effects [121]. The heterogeneity of tumors in space and time and of cells, ECM, and vasculature represents one of the most significant obstacles to efficacy of PDT, and the HFB model is the only one to reproduce it in terms of nutrient gradients and perfusion [122]. ICG diffusion is restricted to the tumor volume in a heterogeneous TME, resulting in resistance to hypoxic nuclei but maximizing perfusion results in apoptosis being increased [123]. Bismuth/manganese HCICG nanoplasty systems are made with the use of heterogeneity to increase their activity, reaching high loading capacities of ICG and penetration, and, consequently, filling tumors without resistance in living organisms [124]. This has clinical implications on heterogeneity mapping pre-PDT using NIR imaging-based biopsies with HFB as a testing host of the personalized dose [125]. Altogether, the findings of the HFB model provide solid evidence for the transition between preclinical work and clinical work with an emphasis on selective toxicity of ICG-PDT as one of the benefits, reducing the risk of adverse effects and low systemic potential of long-term safety, as well as providing methods to overcome heterogeneity using nanoparticles and therapy combinations. Such associations facilitate the speed of translation, which could be used to enhance the possibility of survival in resistant tumors. It is important that these insights be validated in further Phase II/III clinical trials with a longer-term endpoint such as progression-free survival.

Multiomics approaches can reveal molecular signatures of PDT response or resistance, enabling personalized therapy. Ethical issues such as equitable access to advanced therapies, patient education, and rigorous safety assessment of natural compounds are critical to avoiding inequalities in healthcare. The differential sensitivity of MCF-7 cells to ICG-PDT, combined with the preservation of normal cell viability at lower concentrations, suggests a promising therapeutic index for ICG-based therapies. Moreover, cell density-dependent effects highlight the need to consider tumor characteristics such as cellularity when designing PDT protocols. The therapeutic potential of ICG, confirmed by its fluorescent properties, further supports its use in image-guided therapies, where real-time monitoring can increase treatment precision. ICG-PDT, supported by nanotechnology, synergistic combinations and multimodal therapies, shows high potential for selective elimination of MCF-7 cells. However, translating these strategies into clinical practice requires further in vivo studies, optimization of dosimetry, and addressing the issues of tumor heterogeneity and hypoxia, positioning PDT as a promising treatment for breast cancer with broad oncological potential.

The outlook goes toward combination therapy, nanoparticle carriers of ICG, and streamlined light delivery, which have the potential to enhance much better translational activity, particularly in HFB models where dynamic perfusion and real-time tracking are possible. It is not only that these research directions address the limitations of contemporary approaches but also that they open the possibility of personalized cancer medicine through the combination of PDT with other treatment modalities. The integration of ICG-PDT with other treatment methods is one of the main directions of its development, since it is possible to achieve a synergistic effect, which increases the generation of ROS, overcomes hypoxic resistance, and activates the immune response. To illustrate, the ICG-PDT has been shown to work synergistically with the use of chemotherapy agents like etoposide (VP-16) which have shown greater antitumor efficacy through greater apoptosis and proliferation inhibition in models of lung cancer, with PDT only partially yielding remission. On the same note, the synergistic action of concurrent PDT and chemotherapy on colorectal cancer models underscores the importance of tumor volume reduction of more than 70%, owing to the complementary action of ROS and cytostatic on the HIF-1a signaling pathway [60,121]. When used in combination with ICG-PDT with ferritin nanoparticles, not only does the removal of tumor antigens achieve better immunogenicity, but it also promotes abscopal effects by targeting T lymphocytes located in remote metastatic foci, which, in particular, is a promising opportunity in HFB models, where the release of tumor antigens by perfusion can be used to manipulate the TME [126]. A new method that employs magnetic microbubbles in combination with ICG-conjugated liposomes incorporates PDT and ferroptosis and photothermal energy (mPTT) which results in the synergistic destruction of cancer cells in hypoxic environments, which may be applied to potentially employ the method against recalcitrant solid tumors. In addition, the ICG-ALA (5-aminolevulinic acid) complex augments PDT by boosting the synthesis of protoporphyrin IX, which enhances treatment efficacy of skin and gastrointestinal tumors by reducing the amount of light required and decreasing the toxicity [7,126]. Alternatives like ICG-PDT with tirapazamine (TPZ) in polydopamine nanoparticles (PDA) take advantage of the hypoxia of the TME to release drugs selectively, tripling the efficacy of xenograft models and opening the prospect of adapting HFBs to test long-term responses. Perftoran (an oxygen emulsifier) studies have revealed that ICG-PDT, when combined with perftoran, regulates the expression of hypoxia-related microRNAs (HypoxamiRs), making it phototoxic to lung cancer cells. Further research in this area should be on clinical trials of these combinations but must be combined with immune checkpoint inhibitors to achieve maximum results in a heterogeneous TME [59,127]. Stability of ICG under physiological conditions and its rapid clearance and low tumor specificity are some weaknesses that impede the use of ICG in PDT. The solution can be provided by advanced nanoparticle carriers, which have great stability, bioavailability, and targeting, allowing for deeper penetration of the TME, as is simulated in 3D models [126]. Nanoparticles of ferritin loaded with ICG exhibit great selectivity to the transferrin receptors in cancer cells, which amplifies PDT and a robust immune response with an 80% tumor reduction in in vivo models. Liposomes coated with chitosan oleate and ICG enable controlled release under the influence of NIR light, increasing the effectiveness of PDT in prostate cancer by modulating the pH of the TME [128]. Those N-CQDs serve as ICG carriers, and in addition to dye stabilization, they allow for simultaneous cellular imaging, which is important in HFBs—as was demonstrated, N-CQDs showed a two-fold enhancement in ROS generation and decreased hypoxic resistance [129]. Photothermal therapy combined with PDT is synergistic in deep tumors with the use of magnetic polydopamine nanoparticles packaged in calcium carbonate with ICG, in which the biomimetic erythrocyte membrane-based carrier system is used to evade macrophage phagocytosis [128,130]. ICG-functionalized glucose-sensitive nanohydroxyapatite (nHA) demonstrated encouraging outcomes in liver cancer, as tumor accumulation increased by three times, which implies that it can be used in therapies of GLUT1 receptors in a hypoxic TME [131]. Sequential chemotherapy can be performed using hybrid systems, e.g., mesoporous silicates (MSNs) with slow release of doxorubicin following PDT with ICG, with a delay of 8 h being the best. Intelligent carriers sensitive to the TME (e.g., pH, enzymes) should be studied in the future, and they should be combined with HFB models to make the doses optimal and systemic toxicity minimal [132]. TME heterogeneity and tissue absorption limit light delivery NIR in ICG-PDT, giving rise to non-uniform PS activation. Optimization also incorporates the creation of fiber-optic carriers and modulated lasers and image-guided systems, which enhances penetration and precision [133]. Gantrez AN-139 polymer with ICG has been shown to provide improved diffusion in the dermal layers and 50% higher efficacy of PDT in skin cancer models using Gantrez AN-139 polymer with ICG. ICG-Liposomes allow for effective growth inhibition of the tumor and light fluence is optimized at 50 J/cm^2^ to reduce thermal effects [134]. Glycyrrhetinic acid and RGD dual-targeted liposomes in liver cancer integrate PDT with chemotherapy and maximize delivery of light through fluorescence imaging, achieving a 30 percent dose reduction without affecting efficacy [135]. TME-responsive nano-biomimetic systems enhance hypoxia and ICG penetration to allow for the controlled delivery of NIR light to synergistically enable PDT/PTT. Nanosystems with TAT peptides lead to high uptake of ICG in cells under light exposure, which is better than regular cancer therapy, as it increases efficacy 2-fold [42,136]. Fiberoptic perfusion can also be optimized in HFBs, which can then be used to test adaptive AI-based algorithms of real-time symmetry. The future of ICG-PDT, featuring an increased focus on therapeutic combinations, improved nanoparticle carriers, and optimal light delivery, is likely to transform the field of oncology. The combination of these innovations with 3D HFB models will make the process of personalized protocol development faster, reducing resistance to TME and improving clinical outcomes. These approaches will need to be validated with additional clinical trials, such as Phase II/III trials. Also, this in vitro research finding needs to be tested in a living organism to show in vivo limitations and prove it works and is reliable. In vivo studies are complex and costly, but they are crucial to confirm that results seen in vitro are applicable to a whole, complex biological system. Without this validation, our discovery may not reach real-world effectiveness.

## 4. Materials and Methods

### 4.1. Three-Dimensional Cell Culture

Human breast cancer cells (MCF-7) were obtained from ATCC (Manassas, VA, USA) and grown in Dulbecco’s Modified Eagle Medium (DMEM) purchased from ThermoFisher Scientific (Waltham, MA, USA), enriched with 5% FBS, L-glutamine (2 mM), sodium bicarbonate (15 g/L), sodium pyruvate (10 mM), penicillin (100 IU/mL), streptomycin (100 µg/mL), and gentamicin (50 µg/mL). For 3D culturing, cells were introduced into a hollow fiber bioreactor (HFB; FiberSystem Cell Inc., Frederic, MD, USA), which featured microporous fibers that mimicked the in vivo microenvironment by enabling nutrient exchange and waste removal. To support cell adhesion and ECM-like interaction, the internal surface of the fibers was pre-treated with a collagen solution (1 mg/mL in PBS). Phosphate-buffered saline (PBS) was supplied by Amresco Inc. (Solon, OH, USA), and additional FBS was obtained from Invitrogen (Carlsbad, CA, USA). This setup facilitated the development of spheroid tumor-like aggregates over a 60–120-day period, during which the culture medium was refreshed weekly. In a hollow fiber bioreactor system, flow rates could vary from 0.5 to 5 mL/min, with a typical range of 1 to 180 mL/min. Oxygenation was controlled through gas-permeable membranes, with 5–10% CO_2_ often used to regulate pH. Initial cell densities were 1000, 10,000, 100,000, 1 × 10^6^ and 1 × 10^9^. The hollow fiber bioreactor (HFB, FiberSystem Cell Inc., Frederic, MD, USA) contained a specific fiber to allow cells to grow on its surface. In our study we used one fiber with 0.1 m diameter pores. Nourishing elements and waste were delivered in a controlled manner through the fiber’s pores. In this manner MCF-7 cells growing originally in suspension built up a solid 3D tumor. The media flow rate was maintained at 14 mL/min and controlled by a peristaltic pump. As the cells proliferated within the fiber scaffold, their morphology was monitored via phase-contrast microscopy (MicroCap v1.0, ESPA Systems Co., Ltd., Espoo, Finland, 200× magnification), and viability was determined using the trypan blue exclusion method from Sigma-Aldrich (Allentown, PA, USA). Additional labware included precision microscope slides (Corning Inc., Corning, NY, USA), hemocytometers (Hausser Scientific, Horsham, PA, USA), 29 mm glass-bottom dishes with 10 mm microwells (In Vitro Scientific, Sunnyvale, CA, USA), and 96-well round-bottom plates (Corning B.V. Life Sciences, Corning, NY, USA). Sterile vials and purified water were provided by Nalgene (Rochester, NY, USA) and U.S. Filter Corporation (Vineland, NJ, USA), respectively. Cell density was quantified manually using a hemocytometer. Cells were enzymatically detached with a 4% trypsin solution and transferred into 2.6 mm diameter glass capillaries (7 mL volume) for further treatment. These capillaries were maintained in an MRI-compatible incubation chamber under controlled air/CO_2_ perfusion. At least three experiments were conducted for each assay.

### 4.2. Treatment Protocol

Approximately ~10^9^ cells/mL were treated on culture plates and placed for 72 h incubation with concentrations of 0, 1, 10, 100, 500, or 1000 μM ICG [88,137,138] manufactured by Carl Roth (Karsruhe, Germany) at 37 °C in the dark. Unbound PS was then washed out, and cells were incubated in fresh medium for 24 h. Photoactivation was achieved using the diode laser LED Diall IP54 0203H (Warsaw, Poland). Cells were irradiated in glass-bottom dishes under a 780 nm light dose, which was appropriate for ICG activation. Later the singlet oxygen concentrations were measured, and the optimal concentration was chosen. We recognize that the solvent plays a significant role in ICG’s behavior, with aggregation being more pronounced in water compared to ethanol or protein-bound environments like blood or plasma [79]. ICG absorbs light in the near-infrared range, which allows for deeper light penetration of up to several centimeters into tissues, compared to photosensitizers that absorb in the visible light range. This makes ICG more effective in treating deeper lesions, such as atherosclerosis or deep infections, without the need for invasive procedures. ICG dissolves in water, which makes it easier to use compared to lipophilic porphyrins. In addition, ICG has low systemic toxicity, rapid elimination from the body by the liver and kidneys within minutes to hours, and minimal side effects, unlike many other photosensitizers (e.g., porphyrins) which exhibit high phototoxicity, accumulation in the skin, and long-lasting photosensitivity reactions [139,140,141,142,143,144,145,146,147]. Our study accounts for these factors by testing ICG across a range of concentrations and solvents to better understand its photophysical properties and inform its application in both research and clinical contexts. While the higher concentrations are not directly translatable to clinical use, they are relevant for specific in vitro or specialized in vivo applications, such as fluorescence imaging with albumin, as noted in prior studies [78].

### 4.3. Photodynamic Therapy

ICG was incubated for 72 h at 37 °C in the dark. After removing the PS and washing with PBS (EURx Molecular Biology Products, Gdańsk, Poland), the treated cell cultures were irradiated with the diode laser LED Diall IP54 0203H. All irradiations were performed at room temperature at 25 degrees Celsius. The light was emitted isotropically, and the sample was illuminated from 20 cm. We used a nominal power of 400 W and an optical efficiency of 50%, and the effective optical output power of the lamp was 200 W. Under these conditions, the light energy was distributed over the surface of a sphere with a radius of 20 cm, resulting in a total surface area of approximately 5.027 cm^2^

A = 4πr^2^ = 4π (20)^2^ = 400π ≈ 5.027 cm^2^

Given that the illuminated sample area was 1 cm^2^, the power density (irradiance) at the sample surface was calculated as follows:

$$I\;=\;\frac{P}{A}=\frac{200\;W}{5.027\;{cm}^{2}}\;\approx \;0.0398\;W/{cm}^{2}$$

With an exposure time of 15 min (900 s), the total light energy dose (fluence) delivered to the sample surface was as follows:

H = I × t = 0.0398 W/cm^2^ × 900 s ≈ 35.8 J/cm^2^

### 4.4. Cell Viability and Cell Concentration Count

Viability assessments were conducted via trypan blue staining from Sigma-Aldrich (Allentown, PA, USA), with manual cell counts performed using a hemocytometer (Hausser Scientific, Horsham, PA, USA). A 20 μL aliquot of the cell suspension was transferred into an Eppendorf tube, and 380 μL of Muse^®^ Cell Count & Viability reagent (Luminex, Austin, TX, USA) was added. The mixture was incubated at room temperature for 5 min. Cells were then counted (cells/mL) using the Guava^®^ MUSE^®^ Cell Analyzer (Cytek Biosciences B.V., Amsterdam, The Netherlands).

### 4.5. Spectroscopic Measurements

We used FluoTime 300 “EasyTau” (PicoQuant, Berlin, Germany) a fully automated fluorescence spectrometer to measure fluorescence lifetimes and fluorescence spectra. Singlet oxygen studies are typically conducted using steady-state and time-resolved phosphorescence measurements, detecting emission around 1270 nm.

**Figure 10:**
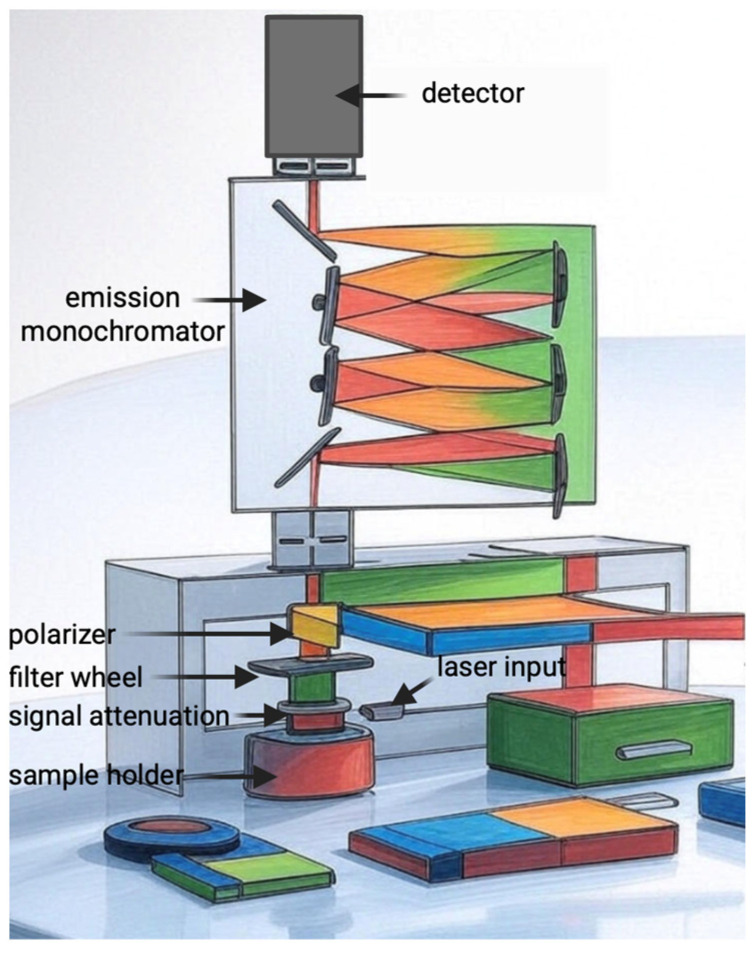
Illustration of the mechanism of FluoTime 300 “EasyTau”.

The measurement protocol was carried out in accordance with the manufacturer’s (PicoQuant) information, using the FluoTime 300 “EasyTau” (Figure 10) system, which is a fully automated spectrometer for fluorescence lifetime measurements, equipped with options for phosphorescence measurements, including singlet oxygen emission in the NIR range. We used a picosecond pulsed diode laser as the ICG excitation source. The emission was collected at an angle of 90° using a NIR-sensitive detector with a monochromator. The EasyTau 2.3. software (PicoQuant, Berlin, Germany) automated the setup, i.e., wavelength selection, slit adjustment (10 μm–4 mm), spectrum acquisition, and kinetics. The data was analyzed in FluoFit with exponential fitting and quality assessment. To optimize sensitivity and minimize noise, the PMT detector was thermoelectrically cooled to reduce dark counts. We used long-pass filters (scattered light rejection 1:10^5^–1:10^10^) and overload protection. The monochromator was set to 1270 nm with a resolution of ≤0.1 nm, NIR-optimized gratings. The sample was placed in a 1 cm cuvette in a multifunctional chamber at a temperature of 15 °C. The entire setup was controlled by software—there were no manual adjustments of the optics during measurement.

### 4.6. Statistical Analysis

Data are reported as mean ± standard deviation. Differences levels in MCF-7 cells and healthy cells were calculated by Student’s *t*-test at *p* < 0.05, using Statistica 13.3 software (TIBCO Software Inc., Palo Alto, CA, USA). The data were presented through Microsoft^®^ Excel.

## 5. Conclusions

PDT with ICG exhibits strong, light-dependent, and selective cytotoxicity toward MCF-7 breast cancer cells while sparing MCF-10A normal breast epithelial cells in 3D culture. Cell viability assays with and without laser irradiation confirmed that cytotoxicity is strictly phototoxicity-dependent, with dark controls showing negligible cell death (<8%) across all tested ICG concentrations (1–1000 μM). Under illumination, MCF-7 viability decreased dose- and cell-density-dependently, reaching >90% cell death at 100–1000 μM ICG, whereas MCF-10A remained largely unaffected (>80% viability). The t-statistics ranged from −2.55 to −14.56, reflecting increasing differences at higher ICG concentrations and seeding densities. These findings highlight the selective cytotoxicity of ICG-PDT against MCF-7 breast cancer cells in a 3D culture system, supporting its potential for cancer therapy research. Detection of singlet oxygen phosphorescence at 1270 nm (0.6 a.u.) and sustained ICG fluorescence (18–20 a.u., 800–820 nm) verified efficient ROS generation upon irradiation. These results establish ICG-PDT as a highly selective, phototoxicity-driven modality for breast cancer cells in vitro. Although the 3D model better mimics tissue geometry than 2D cultures, its in vitro nature limits direct translation to in vivo tumor microenvironments. Future studies should validate its selectivity and efficacy in animal models and explore combinations with chemotherapy or immunotherapy to enhance therapeutic outcomes and advance clinical translation.

## Data Availability

The original contributions presented in this study are included in the article. Further inquiries can be directed to the corresponding authors.

## Abbreviations

The following abbreviations are used in this manuscript:

PDT	Photodynamic therapy
ROS	Reactive oxygen species
ICG	Indocyanine green
BC	Breast cancer
NIR	Near-infrared
ER	Estrogen receptors
PR	Progesterone receptors
